# Comparative Studies of the Effects of Egg Yolk, Oats, Apple, and Wheat Bran on Serum Lipid Profile of Wistar Rats

**DOI:** 10.5402/2013/730479

**Published:** 2012-12-12

**Authors:** J. O. Omole, O. M. Ighodaro

**Affiliations:** Biochemistry Laboratory, Lead City University, Ibadan, Nigeria

## Abstract

Excess consumption of egg especially its yolk has been implicated in hyperlipidaemia (high level of cholesterol and triglyceride in the blood). Conversely, soluble dietary fibers, probably due to their ability to bind free lipid molecules, appear to play an important role in protecting against hyperlipidaemia. This study sought to evaluate the comparative effects of selected sources of fibers: apple, oats, and wheat bran, on serum lipid profile in physiologically normal Wistar rats. Twenty rats were used for the study and were randomized into four groups, with each containing five animals (n  =  5). A group which serves as control was fed with egg yolk while the other three groups were fed with apple, oats, and wheat bran, respectively. After two weeks of feeding, the animals were fasted overnight and blood samples from the retro-orbital sinus of the eye were collected for analyses of lipid profile. The results obtained showed that the group fed with oats had the lowest level of total cholesterol (82.9 ± 1.8 mg), low density lipoprotein (LDL) cholesterol (49.3 ± 1.4 mg), and triglycerides (TG) (75.1 ± 1.7 mg), as well as the highest level of HDL cholesterol (33.9 ± 0.9 mg). On the contrary, the group fed with egg yolk showed the highest level of total cholesterol (117.1 ± 4.4 mg), LDL cholesterol (96.4 ± 1.5 mg), and triacylglyceride (109 ± 2.6 mg), as well as the lowest level of HDL cholesterol (18.5 ± 0.9 mg). There was no significant difference (P  <  0.05) between oats and apple in their effects on blood lipid profile of Wistar rats. Wheat bran, being an insoluble dietary fibre, had less significant (P  <  0.05) effect on the blood lipid profile when compared to oats and apple. Findings from this study may assist physicians and dieticians in recommending appropriate diet for individuals desiring to normalize their blood lipids levels.

## 1. Introduction

Cardiovascular diseases and related disorders are a major cause of mortality both in men and women all over the world [1]. They are commonly characterized by high levels of total cholesterol, triglyceride, and low density lipoprotein (LDL) cholesterol in the blood. Large amount of triglyceride and total cholesterol, more importantly LDL cholesterol in the blood, is often associated with the etiology of cardiovascular diseases and is seen as primary risk factors [2]. High level of lipids in the blood has been associated with hypertension, stroke, and lipid peroxidation [3]. Epidemiological studies support the view that consuming diets rich in soluble fibers (fruits, grains, nuts, and vegetables) reduce the incidence of chronic diseases such as cardiovascular disorders, obesity, and diabetes [4]. A significant correlation between consumption of fibers and serum concentration of lipids has been noted [5]. Meals rich in fiber have been associated with the propensity to reduce the risk of developing cardiovascular diseases and related disorders. Apples, oats, and wheat bran are food products with high fiber content [6] and are likely to reduce the total cholesterol, TG, and LDL cholesterol as well as possibly increase HDL cholesterol in the blood, a condition that lowers the risk of cardiovascular diseases and attendant mortality [5, 7]. There is an inverse correlation between HDL cholesterol and cardiovascular disorders, the higher the HDL cholesterol in the blood the lower the risk of cardiovascular disorder and vice versa. Conversely, LDL cholesterol and TG have direct correlation with susceptibility to cardiovascular disorders. The more these lipids are in the blood, the more prone an individual is to cardiovascular diseases, atherosclerosis, and hypertension. The present study therefore sought to compare the antilipidemic and anticholesteremic effects of oat, apple, and wheat bran in physiologically normal Wistar rats.

## 2. Materials and Methods

### 2.1. Materials

Twenty (20) Wistar rats of opposite sex were obtained from a local breeder in Ibadan, southwest of Nigeria. Egg yolk was obtained by separating the yolk from the albumin and dried in a hot air oven at 80°C for 3 hours to constant moisture content. Healthy apples, canned oats, and wheat bran were purchased from appropriate commercial centers in Ibadan, southwest of Nigeria.

### 2.2. Experimental Design

Twenty (20) male and female Wistar rats weighing between 150 and 160 g were randomly assigned to four groups (A, B, C, and D), *n* = 5. They were housed in individual cage and fed with grower's mash for two (2) weeks after which the rats in different groups were fed as follows for another two weeks. Rats in groups A, B, C, and D were fed with apple, oats, wheat bran, and egg yolk, respectively. Group D serves as negative control (diet without fiber). The daily amounts of food intake by the rats in all the groups were determined and their body weights were measured on weekly basis. After the last food treatment, the rats were fasted for 12 h; blood samples were collected from the retro orbital sinus of the eye by ocular puncture into nonheparinised tubes and allowed to clot at room temperature for 30 min. The blood samples were then centrifuged at 3,000 rpm for 15 min and the serum obtained in each case was used for lipid profile analysis. 

### 2.3. Biochemical Analyses

The concentrations of high density lipoprotein cholesterol, low density lipoprotein cholesterol, trilglyceride, and total cholesterol were determined using commercial kits from Randox Laboratories, United Kingdom. The principle underlining each assay is given below.

#### 2.3.1. Determination of Total Cholesterol

Cholesterol is determined after enzyme hydrolysis and oxidation. The indicator quinoneimine is formed from Hydrogen Peroxide and 4-aminoantipyrine in the presence of phenol and Peroxidase.

#### 2.3.2. Determination of HDL Cholesterol

LDL cholesterol and VLDL cholesterol are precipitated from serum by the action of a polysaccharide in the presence of divalent cations, after which the HDL cholesterol present in the supernatant is determined.

#### 2.3.3. Determination of LDL Cholesterol

LDL cholesterol is determined as the difference between total cholesterol and cholesterol content of the supernatant after precipitation of the LDL cholesterol fraction by polyvinyl sulphate (PVS) in the presence of polyethylene glycol monomethyl ether.

#### 2.3.4. Determination of Triglycerides

Triacylglycerides are determined after enzymic hydrolysis with lipases. The indicator is a quinone imine formed from Hydrogen Peroxide, 4-aminophenazone and chlorophenol under catalytic influence of Peroxidase.

## 3. Results and Discussion

The mean gains in body weight and food intake of the experimental rats at the end of four weeks are shown in Table 1. Rats fed with egg yolk, oats, and wheat bran increased in body weight by 32.6 28.6 and 25.7 g, respectively, while those fed with apple reduced in body weight by 24.1 g. The relative higher gain in body weight of rats fed with egg yolk is probably due to the high fat content of egg yolk.

 Fruits are known to help in body weight reduction through ease of excretion [8]. This tends to explain the loss in body weight observed in rats fed with apple in this study.

Animals fed with oats consumed the highest amount of food, and those placed on with wheat bran consumed the lowest amount of food. Similar quantities of food were consumed by animals in the control group fed with egg yolk and those fed with apple (Table 1). The palatability of the diets obviously affected the rate of consumption of each food and partly accounts for the trend in the final body weights of the animals. 

Table 3 presents the serum lipid profile of rats fed with different diets. As anticipated, the group of rats fed with egg yolk expressed the highest levels of LDL cholesterol (96.4 mg/dL), total cholesterol (117.4 mg/dL), and triglycerides (109.8 mg/dL) as well as the lowest level of HDL cholesterol (18.5 mg/dL). Egg yolk is rich in cholesterol (117.1 mg/dL) and its involvement in the incidence of cardiovascular diseases and atherosclerosis is popular [9]. On the contrary, rats fed with oats recorded the lowest level of total cholesterol (82.9 ± 1.8 mg), low density lipoprotein (LDL), cholesterol (49.3 ± 1.4 mg), and triglycerides (TG) (75.1 ± 1.7 mg), as well as the highest level of HDL cholesterol (33.9 ± 0.9 mg). 

There was no significant difference (*P* < 0.05) between oats and apple in their effects on blood lipid profile of Wistar rats, with the chief constituent of these fibers being pectin and responsible for the hypocholesterolemic effect [10, 11]. Rats fed with apple exhibited increased level of triglycerides (96.9 mg/dL) but exhibited lower levels of LDL cholesterol (57.9 mg/dL) and total cholesterol (88.5 mg/dL) when compared with rats fed with oats egg yolk. Lipogenesis may have accounted for the high triglycerides level observed in rats fed with apple when compared with those fed with oats and wheat bran [8]. The results observed with oat and apple in this study corroborate previous reports [6, 7].

Wheat bran, though has the highest amount of crude fiber (9.9%) (Table 2), had the least effect in improving the lipid profile of rats when compared to oats and apple. The reason for this is obvious in that wheat is chiefly composed of cellulose and lignin which are insoluble dietary fibers [11]. This observation agrees with the report of Jenkins and his colleagues, who reported in one of their studies [9] that wheat bran did not have any significant effect on the serum lipids of individuals fed with it.

Soluble dietary fibers have been demonstrated to be beneficial in the management or treatment of diabetes and cardiovascular disorders [5]. The actual role of these fibers in the entire process is unclear. Possibly, foods rich in soluble dietary fiber either reduced the quantity or facilitate the elimination of other foods which may be risk factors for these diseases. Moreover, diets that are high in fiber tend to be low in energy and these diets can be useful in the control of body weight [12, 13], a critical factor in individual's susceptibility to hyperlipidamia related disorders. 

## 4. Conclusion and Recommendation

Overall, feeding of oats and apple to rats significantly improves the serum lipid profile in this study. Consumption of foods rich in soluble dietary fibers such as oats and apple is highly encouraged. Dietary fiber is found only in plant foods such as fruits, vegetables, nuts, and grains. Milk, meat, and egg do not contain dietary fibers [14]; hence their intake should be minimized especially among adults. Moreover, excessive processing of fiber foods should be avoided. The removal of seed coat, peel, or hull reduces the fiber content of foods. For instance whole tomatoes have more fiber than peeled tomatoes; likewise, whole wheat bread contains more fiber than white bread [15].

## Figures and Tables

**Table 1:** Food intake and body weight gain of rats fed with oats, apple, wheat bran, and egg yolk.

Diet	Food intake (g/day)	Body weight gain (g/4 weeks)
Egg yolk	31.2 ± 3.1	32.6 ± 1.3
Apple	33.8 ± 2.4	−24.1 ± 0.8
Oats	36.9 ± 1.8	28.6 ± 2.1
Wheat bran	28.4 ± 2.9	25.7 ± 1.8

Values are means of 5 determinations with standard deviation.

**Table 2:** Proximate analysis of apple, oats, and wheat bran.

Diet	Moisture (%)	Protein (%)	Crude fat (%)	Crude fibre (%)	Ash (%)	Cabohydrate (%)
Oats	3.1 ± 0.7	11.3 ± 1.0	1.5 ± 0.6	9.3 ± 2.1	4.3 ± 1.4	70.5 ± 1.8
Apple	4.5 ± 1.2	15.2 ± 1.5	1.3 ± 0.8	7.1 ± 2.7	1.4 ± 0.5	83.1 ± 1.1
Wheat bran	11.2 ± 0.0	15.2 ± 1.5	3.5 ± 1.2	9.9 ± 1.7	5.1 ± 1.4	55.3 ± 2.6

**Table 3:** Blood lipid profile of rats after 2 weeks of feeding with different diets.

Diet	Total cholesterol (mg/dL)	HDL cholesterol (mg/dL)	LDL cholesterol (mg/dL)	Triglyceride (mg/dL)
Egg yolk	117.1 ± 4.4^a^	18.5 ± 0.9^a^	96.4 ± 1.5^a^	109.8 ± 2.6^a^
Oats	82.9 ± 1.8^b^	33.9 ± 0.9^b^	49.3 ± 1.4^b^	75.1 ± 1.7^b^
Apple	88.5 ± 1.1^b^	31.2 ± 1.4^b^	57.9 ± 2.6^b^	96.9 ± 1.4^a^
Wheat bran	91.6 ± 1.3^b^	25.7 ± 1.1^b^	78.8 ±0.9^b^	58.9 ± 2.4^b^

Values are means of 5 determinations with standard deviation. Data in the same column with the same superscript are not significantly different (*P* < 0.05).
